# Supplementary material for: Adducts of Carbon Black with a Biosourced Janus Molecule for Elastomeric Composites with Lower Dissipation of Energy
Source: Polymers (Basel). 2023 Jul 22;15(14):3120. doi: 10.3390/polym15143120 (PMC10383720; doi:10.3390/polym15143120)
Supplement: Supplementary file 1 [file polymers-15-03120-s001.zip › polymers-2462765-supplementary.pdf]

Adducts of Carbon Black with a Biosourced Janus Molecule for Elastomeric Composites with Lower Dissipation of Energy

Federica Magaletti, Fatima Margani, Alessandro Monti, Roshanak Dezyani, Gea Prioglio, Ulrich Giese, Vincenzina Barbera and Maurizio Galimberti

Supplementary material

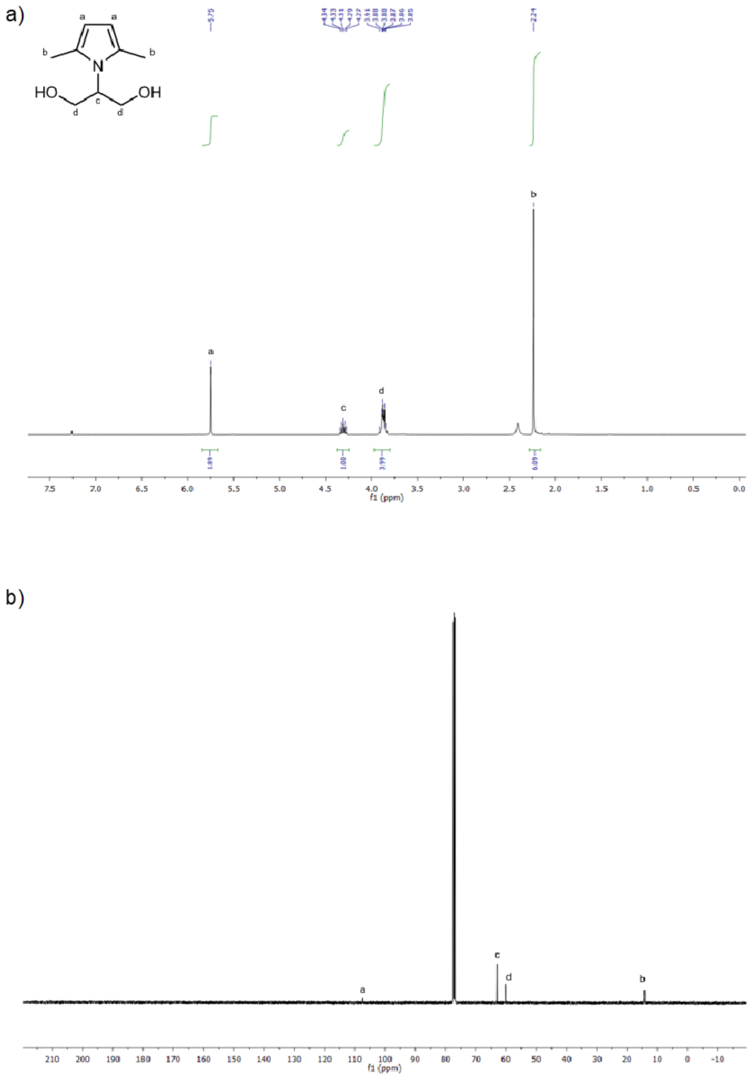

Figure S1. ^{1}H (a, $CDCl_3$, 400 MHz) and ^{13}C (b, $CDCl_3$, 100 MHz) NMR spectra of 2-(2,5-dimethyl-1*H*-pyrrol-1-yl)-1,3-propanediol (serinol pyrrole, SP).

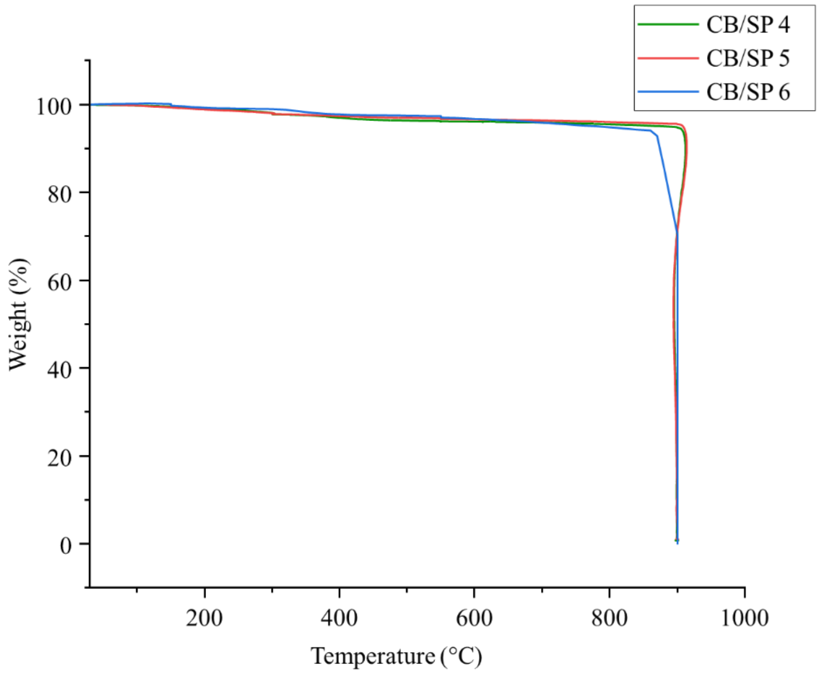

Figure S2 Thermograph from TGA analysis of CB/SP-4, CB/SP-5 and CB/SP-6.

Table S1 Structural parameters from X-ray analysis for pristine CB N326 and CB/SP-6 adduct

Sample	d_{002} (nm)	$D_{\perp}$ (nm)	$D_{\parallel}$ (nm)	$D_{\parallel}$ / $D_{\perp}$	$D_{\perp}$ / d_{002}s
**CB N326**	0.34	1.51	1.54	1.02	4.4
**CB – SP 4**	0.34	1.54	2.12	1.38	4.5
**CB – SP 5**	0.34	1.62	2.03	1.25	4.7

The Bragg's law was used to determine the interlayer distance and the Scherrer equation to estimate the size of the crystallites, orthogonal ($D_{\perp}$), and parallel ($D_{\parallel}$) to the structural layers, through the peak shape analysis of the (002) reflection ($D_{\perp}$) and the (100) reflection. The ratio ($D_{\parallel}$) / ($D_{\perp}$) gives the shape anisotropy.

Table S2 Data from the crosslinking of composites of Table 1[a,b]

	**Silica**	**CB/SP-6**
**M_L [dNm]**	1.95	2.07
**M_H [dNm]**	15.4	19.74
**M_H-M_L [dNm]**	13.45	17.67
**t_{S1} [min]**	2.44	2.12
**t_{90} [min]**	4.02	3.43
**Curing rate [dNm/min][c]**	8.51	13.48

[a] For experimental details see the experimental part

[b] M_L: minimum modulus, M_H: maximum modulus, t_{s1}: induction crosslinking time, t_{90}: optimum crosslinking time,

[c] the curing rate was calculated by means of the following equation:

$$Curing\ Rate = \frac{M_H - M_L}{t_{90} - t_{s1}}$$

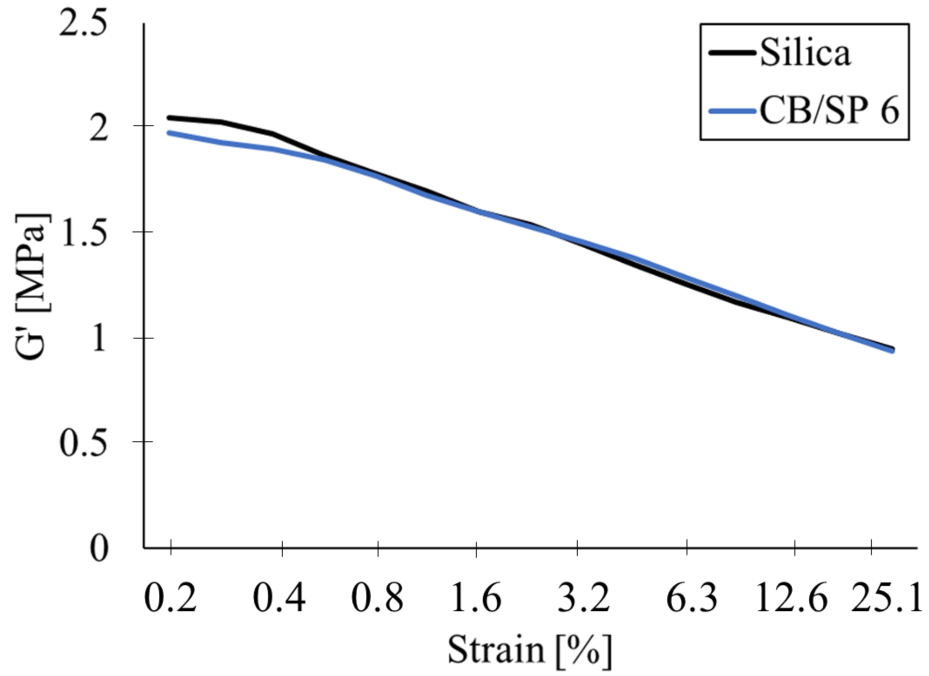

Figure S3. G' vs strain for composites of **Table 1**

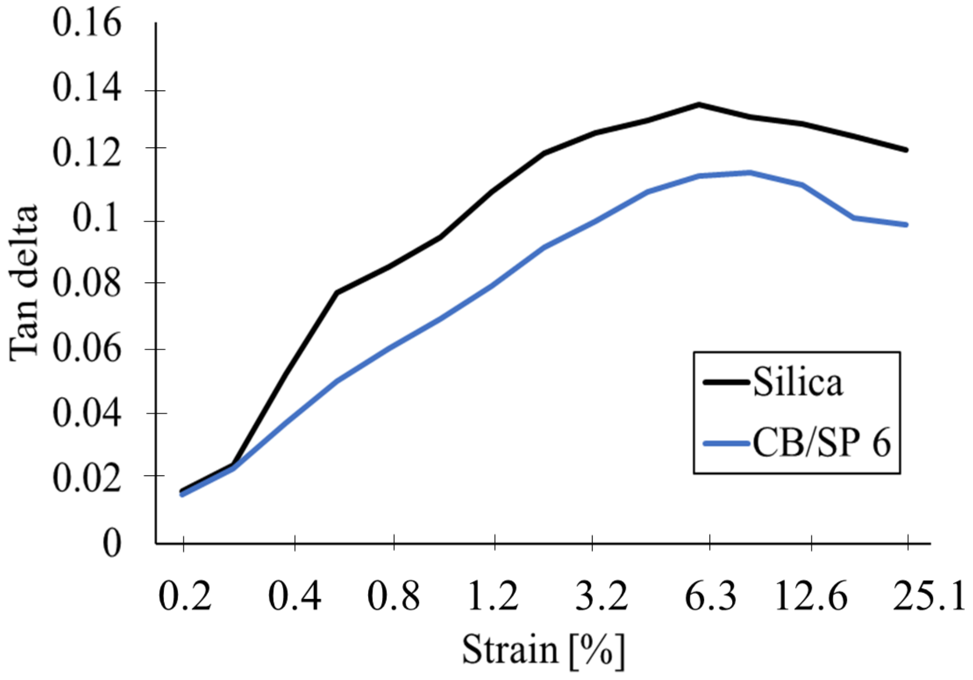

Figure S4. Tan delta vs strain for composites of **Table 1**

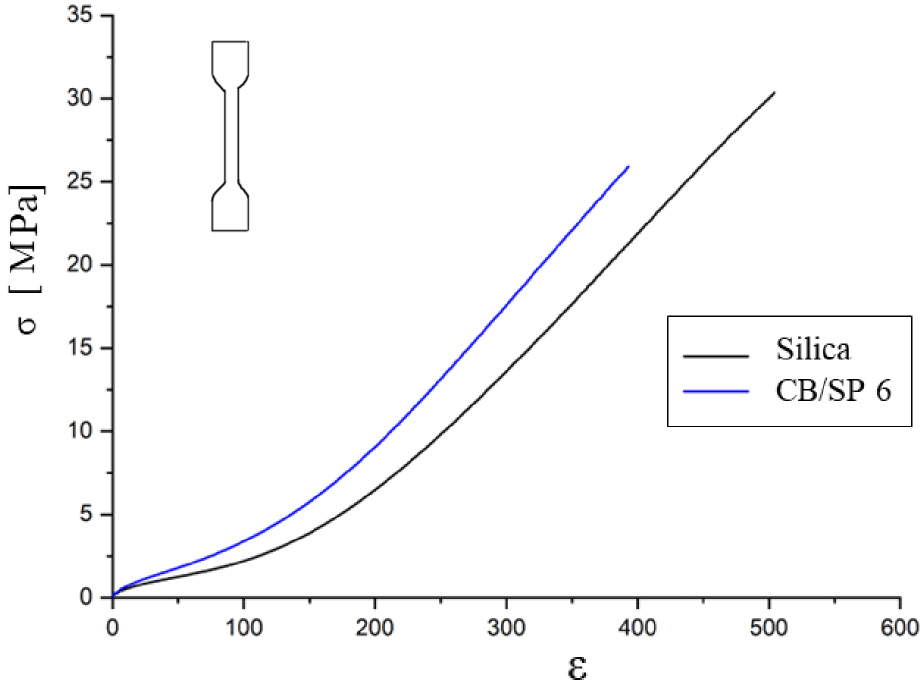

Figure S5. Tensile properties for composites of **Table 1**

Table S3 Data from the crosslinking reaction of composites of Table 2[a,b]

	Silica	CB/SP-4	CB/SP-5
**M_L [dNm]**	3.6	2.75	3.01
**M_H [dNm]**	22.42	21.21	23.29
**t_{90} [min]**	4.26	4.35	4.21
**t_{s1} [min]**	2.33	2.50	2.38
**Curing rate [dNm/min] [c]**	9.75	9.98	11.08

[a] For experimental details see the experimental part [b] M_L: minimum modulus, M_H: maximum modulus, t_{s1}: induction crosslinking time, t_{90}: optimum crosslinking time,

[c] the curing rate was calculated by means of the following equation:

$$Curing\ Rate = \frac{M_H - M_L}{t_{90} - t_{s1}}$$

Table S4. Dynamic-mechanical properties from shear tests of the uncured composites of Table 2.

	Silica	CB/SP-4	CB/SP-5
**$G'_{0.2\%}$ [MPa]**	1.43	1.16	1.15
**$G'_{25\%}$ [MPa]**	0.35	0.30	0.31
**$\Delta G'$ [MPa]**	1.08	0.86	0.84
**$\Delta G'/G'_{0.2\%}$**	0.76	0.74	0.73
**G''_{max} [MPa]**	0.42	0.33	0.31
**$Tan(\delta)_{max}$**	0.45	0.44	0.42

Table S5. Dynamic-mechanical properties from shear tests of the cured composites of Table 2

	Silica	CB/SP-4	CB/SP-5
**$G'_{0.2\%}$ [MPa]**	4.03	3.47	3.84
**$G'_{25\%}$ [MPa]**	1.25	1.18	1.30
**$\Delta G'$ [MPa]**	3.09	2.54	2.74
**$\Delta G'/G'_{0.2\%}$**	0.77	0.73	0.71
**G''_{max} [MPa]**	0.47	0.38	0.41
**$Tan(\delta)_{max}$**	0.21	0.19	0.19

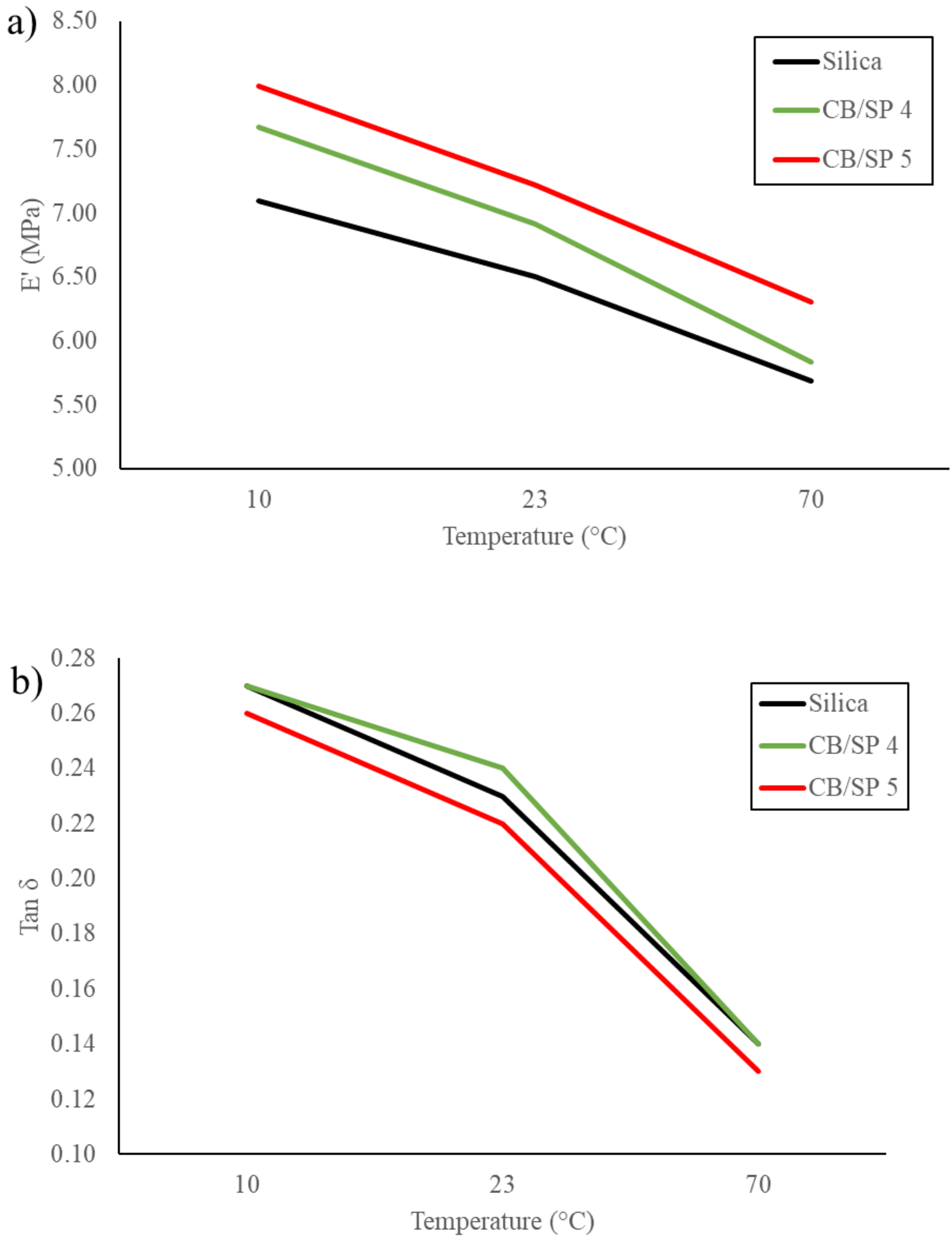

Figure S6 Storage modulus (a) and tan δ (b) curves of IR composites.

Table S6 Tensile properties of composites of **Table 2**

	**Reference**	**CB/SP-4**	**CB/SP-5**
**σ_{50} [MPa]**	1.54	1.69	1.91
**σ_{100} [MPa]**	2.56	2.95	3.48
**σ_{300} [MPa]**	12.60	14.67	16.44
**σ_{300}/ σ_{100}**	4.92	4.97	4.72
**σ_b [MPa]**	24.19	25.39	25.70
**ε_b [%]**	487.89	456.63	436.35
**Energy [MJ/m^3]**	50.18	49.66	49.84

Table S7 Optic microscopy CB dispersion results of IR-based rubber composites

	**Silica**	**CB/SP 4**	**CB /SP 5**
**Filler**	CB	CB	CB
**Filler %**	16	28	28
**N° Aggregates**	10	4	16
**Average diameter [µm]**	16.9	21.1	19.7
**Undispersed filler %**	0.27	0.16	0.32
